# Arm race between Rift Valley fever virus and host

**DOI:** 10.3389/fimmu.2022.1084230

**Published:** 2022-12-22

**Authors:** Xiao Wang, Yupei Yuan, Yihan Liu, Leiliang Zhang

**Affiliations:** ^1^ Department of Infectious Diseases, Shandong Provincial Hospital Affiliated to Shandong First Medical University, Jinan, Shandong, China; ^2^ Department of Pathogen Biology, School of Clinical and Basic Medical Sciences, Shandong First Medical University and Shandong Academy of Medical Sciences, Jinan, Shandong, China; ^3^ Medical Science and Technology Innovation Center, Shandong First Medical University and Shandong Academy of Medical Sciences, Jinan, Shandong, China

**Keywords:** Rift Valley fever virus, innate immunity, interferon, immune evasion, nonstructural proteins

## Abstract

Rift Valley fever (RVF) is a zoonotic disease caused by Rift Valley fever virus (RVFV), an emerging arbovirus within the *Phenuiviridae* family of *Bunyavirales* that has potential to cause severe diseases in both humans and livestock. It increases the incidence of abortion or foetal malformation in ruminants and leads to clinical manifestations like encephalitis or haemorrhagic fever in humans. Upon virus invasion, the innate immune system from the cell or the organism is activated to produce interferon (IFN) and prevent virus proliferation. Meanwhile, RVFV initiates countermeasures to limit antiviral responses at transcriptional and protein levels. RVFV nonstructural proteins (NSs) are the key virulent factors that not only perform immune evasion but also impact the cell replication cycle and has cytopathic effects. In this review, we summarize the innate immunity host cells employ depending on IFN signal transduction pathways, as well as the immune evasion mechanisms developed by RVFV primarily with the inhibitory activity of NSs protein. Clarifying the arms race between host innate immunity and RVFV immune evasion provides new avenues for drug target screening and offers possible solutions to current and future epidemics.

## 1 Introduction

Rift Valley fever virus (RVFV), belonging to the *Phlebovirus* genus of the *Phenuiviridae* family from *Bunyavirales (* 1), is an arthropod-borne virus that affects people and livestock. It was first discovered in 1930 when a fatal infectious disease broke out among sheep in the Rift Valley, Kenya (2). Since the 1950s, the RVF pandemic had regularly occurred throughout Africa (3). The infected area expanded to Yemen and Saudi Arabia in the Arabian Peninsula after 2000 (4, 5). Transmitted by *Aedes* and *Culex* mosquitoes, RVFV spreads in larger geographic ranges due to climate change, making its propagation a possible hazard to non-epidemic countries (6, 7). Instead of mosquito bites, most human cases are caused by contact with infected animal fluids or tissues (8). RVFV causes human diseases including mild flu-like symptoms, hepatitis, retinitis, lethal encephalitis, and hemorrhagic fever, and the overall mortality rate is 0.5 to 1% (9). Pregnant livestock, especially sheep, are highly susceptible to RVFV infection. It generates abortion storms in which almost all pregnant infected animals have miscarriages (10). It also incurs a high mortality rate among newborn lambs (11). Therefore, RVFV infection has severe economic and human health costs. RVFV is now classified as a Category A disease by the National Institute of Allergy and Infectious Diseases (NIAID) and the National Institutes of Health (NIH) because of its potential for purposeful aerosol transmission and the absence of FDA-approved antiviral therapies or licensed vaccinations for humans. RVFV is also a select agent by the Centers for Disease Control and Prevention (CDC) and the U.S. Department of Agriculture (USDA).

**Figure 1:**
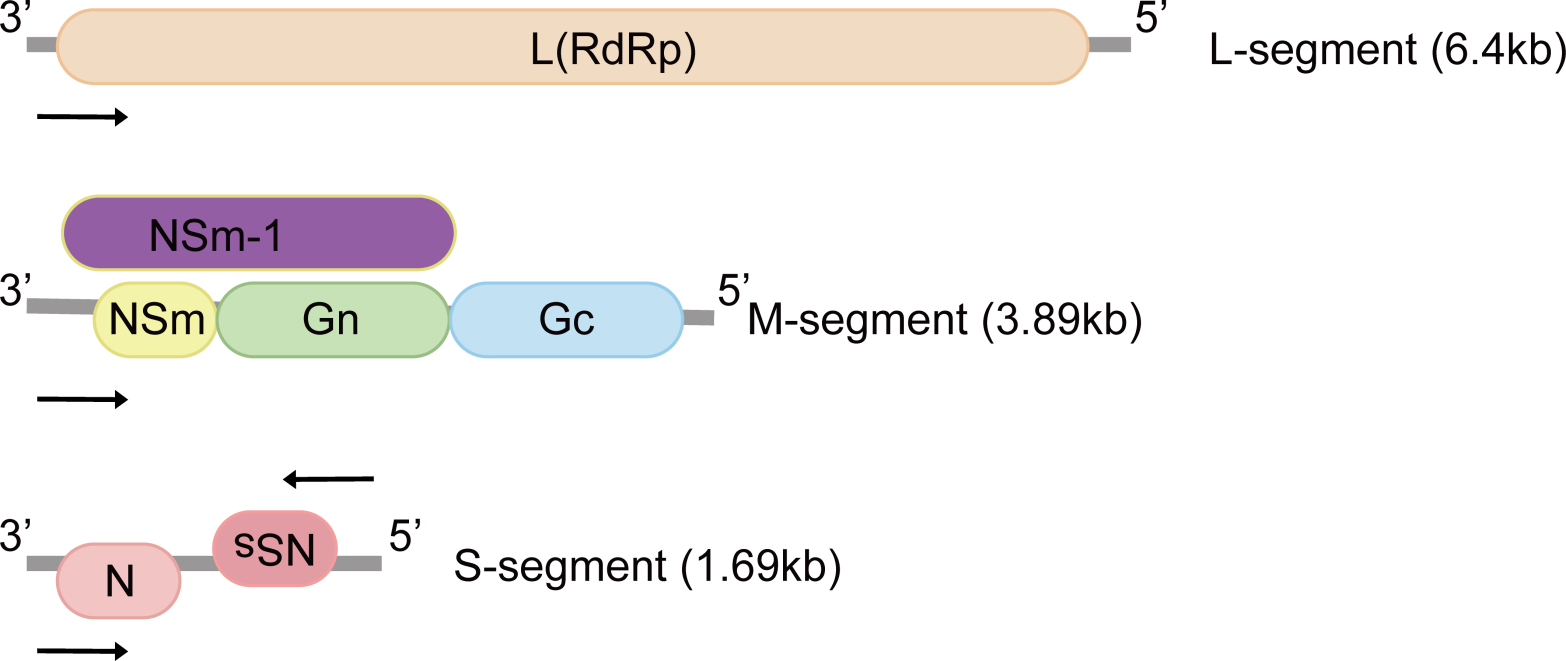
Genomic structure of Rift Valley fever virus. The tripartite negative-sense single-stranded RNA segments are named according to sizes: small (S), medium (M) and large (L), and proteins encoded by each segment are illustrated. The M-segment encodes at least four types of proteins: Gn, Gc, NSm and NSm-1. N and NSs are expressed in an ambisense manner. L, L protein; RdRp, RNA-dependent RNA polymerase; Gn, Gc: glycoproteins; NSm, non-structural protein M; N, nucleocapsid protein; NSs, non-structural protein S.

RVFV genome consists of tripartite negative-sense single-stranded RNA segments. The RNA-dependent RNA polymerase is encoded by the large (L) segment (**Figure 1**). The medium (M) segment encodes the nonstructural protein NSm and envelope glycoproteins Gn and Gc. NSm-1 and NSm-2 are expressed from alternative start codons (**Figure 1**) (12). The nucleocapsid protein (N) and the nonstructural proteins (NSs) are both encoded by the small (S) segment (**Figure 1**). These two proteins are expressed in an ambisense manner, which means the N gene is encoded in the negative-sense genome, and NSs gene is encoded in the positive-sense genome (13, 14).

When host cells detect an RNA virus, a series of complicated innate immune responses are initiated to eliminate the virus, alert cells nearby, and assemble more specialized immune cells to the site of infection. Retinoic acid-inducible gene I (RIG-I), melanoma differentiation factor 5 (MDA5), and Toll-like-receptors (TLRs) are cytosol pattern recognition receptors (PRRs) capable of detecting RNA viruses (15). RIG-I can be recruited to the mitochondria where it associates with the mitochondrial antiviral signaling protein (MAVS) (16). This activated RIG-I/MAVS complex serves as the intersection of multiple innate immune pathways stimulated immediately, particularly the interferon (IFN) and nuclear factor κB (NF-κB) signals (17). However, RNA viruses have advanced procedures to avoid, exploit, or dysregulate these innate immune pathways, which can be seen in chikungunya virus (CHIKV) infection (18). The E1, E2, and nsP2 proteins of CHIKV potently inhibit the activity of MDA5/RIG-I, and nsP2 also suppresses the downstream phosphorylation of signal transducer and activator of transcription 1 (STAT1), blocking the IFN-induced JAK-STAT pathway (18). In this review, we summarize host innate immune responses to RVFV and how they are dysregulated by viral interference, which will offer possible insights into the vaccine and antiviral developments against RVFV.

## 2 Innate antiviral host defense: Interferon response as the crucial step

Interferon is a potent cytokine and a key component of the first line of defense against viral infection (19), which has immunological effects mainly through the direct induction of anti-pathogen molecules that inhibit viral replication (20). There are three types of IFNs involved in antiviral immunity, including IFN-I, IFN-II, and IFN-III. IFN-I and IFN-III share important antiviral properties and are expressed by cells with immunologic and tissue specificity (20, 21).

RNA-triggered intrinsic immunity of RVFV is initiated predominantly by recognition of RIG-I (22). RIG-I consists of a C-terminal domain, a DECH helicase, and N-terminal caspase activation and recruitment domains (CARDs). When cytoplasmic RIG-I is bound with viral RNA, its recruitment to MAVS is activated through the liberated CARDs (23). RIG-I/MAVS complex then catalyzes the combination of TANK-binding kinase 1(TBK1) and inhibitor of κB kinase ϵ (IKKϵ) to phosphorylate and dimerize interferon regulatory factor 3 (IRF3). The phosphorylated dimeric IRF3 could be transported into the nucleus to directly promote IFN-I transcription (24). IFN-I aims primarily to activate the JAK/STAT immune signals in autocrine and paracrine manners, which results in IFN-stimulated genes (ISGs) expression, eliciting subsequent adaptive immune responses (25).

### 2.1 MAVS is crucial for mounting IFN-I response

RIG-I is critical for IFN generation in a TLR-independent way by primary immune cells like macrophages and dendritic cells. That signaling through MAVS protects cells against mortality and mild morbidity during live RVFV mucosal infection (22). RVFV has been emerging as a noticeable neuropathogen (26, 27) and airborne transmission causes severe encephalitis. To understand the precise molecular mechanisms by which RVFV infection is controlled in the brain, a recent study found that microglia, the resident immune cell in the central nervous system acting as macrophages, strongly upregulated transcriptional levels of antiviral immune genes and increased levels of activation markers as well as cytokine secretion. This process was dependent on MAVS rather than TLR3 or TLR7 (28). MAVS-/- mice displayed IFN-I defects and lymphocyte infiltration dysregulation, leading to enhanced susceptibility to RVFV and higher mortality. This study defines a protective role for MAVS in propagating antiviral responses in the brain and suggests that signaling through MAVS may also be required for cerebral functional T and NK cell responses.

### 2.2 Intrinsic antiviral effect of exosomes

Exosomes belong to extracellular vesicles (EVs) and make contributions to cell–cell communication, immunomodulation, as well as infectivity enhancement during viral infections (29). The content of exosomes depends on the cellular origin and the type of infection (30, 31). They are thought to originate from late endosomes and then are secreted into the extracellular environment (32).

Although studies have shown the role of exosomes in viral infections (33), little is known about the mechanisms by which exosome exchanges control the immune response and impact the pathogenesis of RVFV. Researchers generated RVFV-resistant latent clones whose exosomes contain not only normal marker CD63 but also viral RNA and proteins like N and NSs (34). Some of the neighboring recipient cells showed drastically increased apoptosis *via* PARP cleavage and caspase 3 activation. Later, one study revealed how exosomes affect viral production and protect recipient cells in an innate immune manner (35). Exosomes that are purified from RVFV-infected cells carry RNA genome segments, which activate RIG-I to induce IFN-dependent activation of autophagy in naïve recipient cells like monocytes to suppress viral replication and dissemination.

### 2.3 Host cell metabolites and immune response

#### 2.3.1 Polyamine depletion stimulates innate immune signal

To successfully infect a host cell, viruses need cellular metabolites, and there are different ways they can take over these molecules. One of the critical members of these metabolites is polyamines. They are small, positively charged host-derived molecules that play diverse roles in human cells (36), and polyamine-depleted mammalian cells maintain viability without significant toxicity (37). RNA viruses rely on polyamines for replication (38) and a recent study showed that diverse bunyaviruses, especially RVFV, La Crosse virus (LACV), and Keystone virus (KEYV), require polyamines for productive infection (39). Viral noninfectious particles can interfere with productive infection *via* binding cellular receptors or usurping cellular and viral machinery from infectious viruses (40). In polyamine-depleted cells, bunyaviruses produce a large number of noninfectious virions that are indistinguishable from infectious particles, but these particles could disrupt productive infection and stimulate antiviral signaling pathways like the IFN-I pathway. To conclude, polyamine depletion results in the accumulation of noninfectious particles that interfere with viral replication and stimulate innate immune signaling to limit infectivity. Later, researchers investigated how polyamines precisely function in RVFV infection and found that spermidine, a specific type of polyamine, is required for RVFV replication (41). Furthermore, RVFV also relies on polyamines for cholesterol synthesis to complete replication and form progeny virions, including the incorporation of cholesterol in virions (42). It will emphasize a promising method of targeting host polyamines to reduce virus replication.

#### 2.3.2 AMPK inhibits fatty acid synthesis to restrict viral infection

Viruses also manipulate cellular lipids to form complex structures required for viral replication, many of which are dependent on *de novo* fatty acid synthesis (43). For example, envelope formation during viral assembly involves membrane lipid modifications (44). The energy regulator AMP-activated protein kinase (AMPK), which strongly inhibits fatty acid synthesis (45), could restrict infection of RVFV, and it relies on the upstream activator LKB1 (46). AMPK is activated during RVFV infection, leading to the phosphorylation and inhibition of acetyl-CoA carboxylase, the first rate-limiting enzyme in fatty acid synthesis. Therefore, the activation of AMPK both restricts infection and reduces lipid levels. Also, this pathway plays a broad role in the antiviral defense of various arboviruses. Taken together, AMPK is an important component of the host cell innate immune response that provides a novel antiviral therapeutic target associated with the suppression of fatty acid metabolism.

### 2.4 TCF/β-catenin regulates virus-induced IFN-β expression

Production of IFN-β plays a key role in the innate antiviral response. Using genome-wide RNA interference (RNAi) screening, canonical Wnt/β-catenin signaling was found to be an important host pathway during RVFV infection. It can regulate optimal cell cycle conditions and mediate the formation of the TCF/β-catenin complex to promote efficient viral replication (47). β-catenin can be found within a degradation complex associated with GSK-3, the Wnt/β-catenin pathway kinase. Inhibiting GSK-3 increases the amount of β-catenin and promotes its nuclear accumulation. β-catenin interacts with T-cell factor (TCF), rather than IRF3, to form the TCF/β-catenin complex, which can be recruited over the IFN-β promoter and increase the degree of constitutive IFN-β expression in uninfected cells (48). Additionally, raising the level of constitutive IFN-β is capable of conferring an effective antiviral state to naïve cells in order to promote subsequent virus-induced IFN-β expression. In RVFV infection, active TCF/β-catenin complexes are formed and the host Wnt/β-catenin pathway is targeted at the transcriptional and protein levels. NS protein is the major virulent factor to inhibit Wnt/β-catenin signaling by regulating relevant gene expression. Removal of NS protein from RVFV activates the Wnt/β-catenin pathway, forming a TCF/β-catenin complex, and TCF directly upregulates IFN-β expression.

## 3 Viral countermeasure and innate immune evasion

Host cells tend to take immediate measures to limit viral replication and propagation right after being infected, and simultaneously the virus initiates countermeasures to limit the cell’s antiviral responses. This includes suppressing the host innate immune pathway, and directly disrupting host gene expression.

### 3.1 Alternative splicing of RIOK3 during RVFV infection reverses its antiviral and anti-inflammatory effects

Transcriptome studies have revealed that viral invasion could change host splicing patterns (49). A significant post- and co-transcriptional regulatory mechanism known as alternative splicing (AS) affects the expression of more than 95% of the genes in the human genome and increases genetic coding capacity (50). Through its functional relationship with nonsense-mediated decay (NMD) to degrade premature termination selectively, AS enables the creation of structurally varied protein isoforms from a single gene and can help regulate gene expression (51, 52).

**Figure 2:**
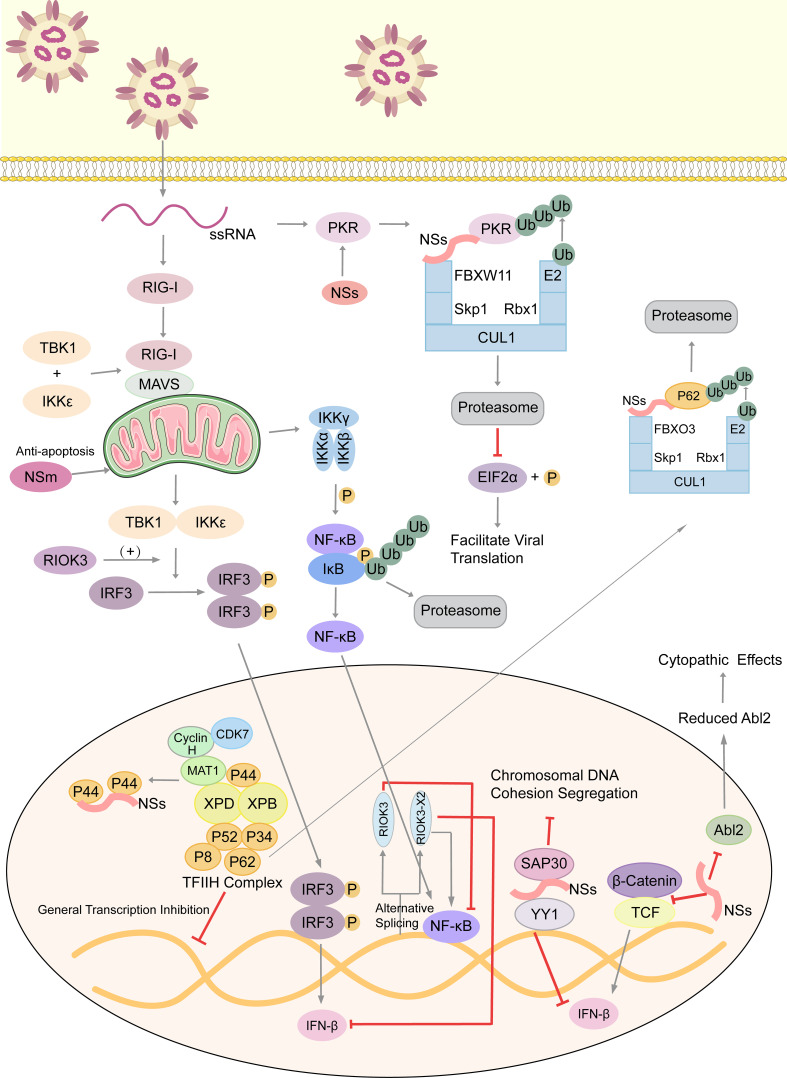
Arm race between RVFV and host, with emphasis on immune evasion by RVFV. RVFV ssRNA is recognized by cytosolic RIG-I. RIG-I associates with mitochondrial MAVS to activate multiple innate immune pathways. The kinase RIOK3 facilitates IFN expression and inhibits the inflammatory response pathway mediated by NF-κB, while the alternative splicing isoform RIOK3 X2 antagonizes these effects. RVFV infection also stimulates the Wnt pathway to produce IFNβ, but the NSs protein inhibits this process. NSs conduct the immune escape from several aspects. Those include inhibiting the aggregation of TFIIH complex to extensively inhibit host transcription, degrading kinase PKR to decrease eIF2α phosphorylation and promote the translation of viral proteins, and forming SAP30-NSs-YY1 co-repressor complex at the IFN promoter to block its transcription. Moreover, NSs affect the formation of cytoskeleton *via* suppressing Abl2 expression, which changes cell morphology and movement. Also, NSs could damage the host chromosomal DNA and disrupt mitosis. NSm, however, plays an anti-apoptotic role in mitochondria. ssRNA, single-stranded RNA; RIG-I, retinoic acid-inducible gene I; MAVS, mitochondrial antiviral signaling protein; TBK1, TANK-binding kinase 1; IKK, inhibitor of κB kinase; IκB, inhibitor of NF-κB; IRF3, interferon regulatory factor 3; TCF, T-cell factor; RIOK3, RIO Kinase 3; TFIIH, transcription factor IIH; CUL1, cullin 1; FBXO3, F-box protein 3; Skp1, S-phase kinase associated protein 1; FBXW11, F-box and WD repeat domain containing 11; Rbx-1, ring-box 1; PKR, protein kinase R; eIF2α, eukaryotic initiation factor 2α; SAP30, Sin3A associated protein 30; YY1, transcription factor Yin Yang 1; Abl2, Abelson murine leukemia viral oncogene 2; P, phosphate group; Ub, ubiquitin.

Atypical RIO Kinase 3 (RIOK3) has been demonstrated to play a significant role in promoting IFN-I production *via* PRR signaling mediated by RIG-I-like receptors to inhibit RVFV propagation (53). However, RIOK3 mRNA expression is distorted shortly after RVFV infection to produce alternatively spliced variants, RIOK3 X2, the truncated protein encoding premature termination codons to act as NMD substrate (54). This alternative splicing of the RIOK3 transcript reduces interferon expression conversely. Splicing factor TRA2-β is the key to regulating RIOK3 splicing isoforms (55). TRA2-β interaction with specific regions of RIOK3 pre-mRNA is essential for constitutive splicing of RIOK3 mRNA and RIOK3’s antiviral effect while lacking TRA2-β increases alternative splicing. TRA2-β mRNA is also alternatively spliced during RVFV infection, leading to a decrease in cellular TRA2-β levels. The roles of RIOK3 and its spliced isoform in both IFN and NF-κB pathways are intriguing (56). RIOK3 negatively regulates the inflammatory response, but RIOK3 X2 reverses the effects, mitigating the IFN response and increasing the inflammatory NF-κB response. Therefore, both RIOK3 and its X2 isoform have particular functions in separate RVFV-induced innate immune pathways (**Figure 2**).

### 3.2 NSs protein: primary virulence factor inducing immune escape

#### 3.2.1 Main functions of RVFV NSs protein

RVFV NSs accumulates in the nucleus and cytoplasm, while nuclear NSs forms a filamentous structure (57). Encoded on the S-segment of the RVFV genome, it is an important virulence factor which could potently suppress the innate immune response (58). NSs binds to Sin3A Associated Protein 30 (SAP30), and through interactions with the transcription factor Yin Yang 1(YY1) protein, the NSs-SAP30-YY1 complex blocks the activation of the IFN-β promoter (59). Viral evasion can occur with the contribution of NSs protein since RVFV lacking NSs is shown to induce abundant IFN-I in mice and no viremia is present (60).

Also, NSs generally inhibits host transcription and facilitates viral translation. Eukaryotic transcription factor IIH (TFIIH) is a general transcription factor for transcriptional initiation by eukaryotic RNA polymerase II and plays an important role in nucleotide excision DNA repair. TFIIH is comprised of ten subunits, including the core complex XPD, XPB, p44, p62, p8, p34 and p52. RVFV NSs could competitively bind to p44 and sequester it from binding with XPD (61). p62 is degraded by NSs in a post-translational way. Under the ubiquitin-proteasome pathway, NSs works as an adaptor protein in the cullin 1-Skp1-FBXO3 E3 ligase complex for p62 degradation (62, 63). These two methods disrupt the recruitment of the TFIIH complex in the nucleus, thus leading to the host transcriptional shutoff (**Figure 2**).

Similarly, RVFV NSs protein enhances the post-translational degradation of dsRNA-dependent protein kinase R(PKR) (64). PKR is a translation-inhibiting protein kinase. It can phosphorylate Eukaryotic initiation factor 2α (eIF2α), and then phosphorylated eIF2α inhibits the translation process. NSs participates in the formation of the E3 ligase complex, which consists of CUL1, Skp1, and FBXW11 (65, 66). This E3 ligase complex promotes the degradation of PKR *via* the ubiquitin-proteasome system as well. In consequence, PKR-mediated eIF2α phosphorylation is blocked and viral translation is facilitated effectively (**Figure 2**) (67).

Because of the genetic similarity of viruses within the *Phenuiviridae* family, NSs is also a key virulence factor of other phenuiviruses and its antiviral immune suppression in those phenuiviruses is worth investing in. NSs of *Dabie bandavirus* (severe fever with thrombocytopenia syndrome virus, SFTSV) has an intriguing mechanism to form granules in the cytoplasm, the inclusion bodies, to entrap factors involved in IFN-induced antiviral responses, like TBK1 and the E3 ubiquitin ligase TRIM25 that is essential for RIG-I activation (68). TBK1 is a critical regulator of not only IFN responses but also the NF-κB inflammatory pathway because it hinders the form of the IKK complex to limit the release and nuclear translocation of NF-κB. The inhibition of TBK1 during SFTSV infection leads to hyper-activation of NF-κB and inflammatory response (69). NSs of SFTSV could induce the cytokine storm, which leads to a high fatality rate of SFTS. Therefore, the different regulatory mechanisms of NS proteins in the innate immune system between RVFV and SFTSV have strong correspondence with their divergent pathogenicity and clinical manifestations.

#### 3.2.2 NSs affects host cell replication

Nuclear abnormalities and a decreased mitotic rate observed in RVFV-infected cells, like micronuclei and lobulated nuclei, are largely because of the chromosomal cohesion and segregation defects (70). NSs filaments accumulating in the nucleus induce canonical DNA damage signaling, including checkpoint kinase 2 (Chk2), ataxia-telangiectasia mutated (ATM), and p53 (**Figure 2**). They also induce cell cycle arrest at the S phase or the G0/G1 phase (71). The SAP30-YY1 complex formed by NSs protein could affect not only IFN-β expression but also the cohesion and segregation of chromatin DNA. Through the SAP30-binding domain, RVFV NSs filaments interact with the pericentromeric major γ-satellite sequence, but not the centromeric minor α-satellite sequence. Also, YY1 could mediate the interaction between the NSs-SAP30 complex and the γ-satellite sequence DNA (70). It is assumed that through NSs-mediated DNA damage, erroneous host cell replication impairs normal tissue development and may contribute to fetal deformity in infected ruminants.

#### 3.2.3 NSs and cytopathic effects

Besides functioning as the main virulence factor counteracting the host innate antiviral response to facilitate viral replication and spread, the role of NSs in RVFV-induced cytopathic effects was investigated (72). Abelson murine leukemia viral oncogene homolog 2 (Abl2) is a key regulator of the actin cytoskeleton, regulating cell morphology and mobility as well as cell-cell and cell-matrix adhesion (73, 74) *via* its tyrosine kinase domain and two filamentous actin binding domains (75). The impact of NSs expression on the actin cytoskeleton was examined when carrying out infections with the NSs-expressing virulent (ZH548) strain, the attenuated (MP12) strain, and the non-NSs-expressing (ZH548ΔNSs) strain, as well as following the ectopic expression of NSs. The upregulation of Abl2 expression in macrophages, fibroblasts, and hepatocytes, which would be identified as a component of antiviral responses, was blocked by NSs expression. In addition, ZH548-infected cells had increased mobility compared to ZH548ΔNSs-infected fibroblasts with substantial alterations in cell morphology, including the loss of lamellipodia, cell spreading, and distortion of adherens junctions. All these phenomena are similar to the ZH548-induced cytopathic effects seen *in vivo*. Taken together, NSs protein affects the actin cytoskeleton of host cells at the transcriptional and cellular levels, and the upregulation of Abl2 expression is proposed to be part of the host strategy to restrict virulence (**Figure 2**).

### 3.3 Anti-apoptotic role of NSm proteins

Like NSs protein, NSm is not essential for viral replication in cell cultures (76). A recent study screened and identified 9 host proteins that putatively interact with RVFV NSm, and three of them (Cpsf2, Ppil2, SNAP-25) are the most promising targets during viral infection (77). RVFV NSm was identified as the first *Phlebovirus* protein that has an anti-apoptotic function (78). The C-terminal region of NSm, which contains a basic amino acid cluster and a putative transmembrane domain, targets itself to the mitochondrial outer membrane to resist apoptosis (**Figure 2**) (79).

In comparison to RVFV arMP-12-infected cells, RVFV arMP-12-del21/384-infected cells which lacked NSm expression caused widespread cell death because of the cleavage of Caspase-3 and its downstream substrate poly(ADP-ribose) polymerase. And the initiator caspases, caspase-8 and -9, were all activated earlier. Further, NSm does not require other viral proteins to prevent cell apoptosis because NSm production prevents the staurosporine(STP)-induced activation of caspase-8 and-9. The P38-MAPK pathway is essential for cell survival, and RVFV NSm could also regulate the p38-MAPK response in mammalian cells (80). The specific host factors involved in the NSm-mediated anti-apoptosis are worth investigating, and whether NSm contributes to reaching a balance with the pro-apoptotic NSs protein is an interesting problem requiring further comparison of various environmental factors and mutual molecular mechanisms.

## 4 Conclusion and perspectives

As one of the most important bunyaviruses, RVFV has been responsible for significant human and ruminant outbreaks that have devastated local economies with increasing mortality and morbidity. Upon viral infection, the innate immune system is activated as the first line of defense. IFN response is induced by intricate upstream pathways. The MDA5 and RIG-I are RIG-I-like receptors (RLRs) sensing foreign RNA in the cytoplasm, which transduce a signaling cascade to induce downstream IFN production and subsequent antiviral responses. Lack of metabolites essential for the viral replication cycle and production of noninfectious particles are also methods against RVFV infection. RVFV evolves to evade immune attacks and in turn impairs cellular functions, which is mainly achieved by the powerful virulent NSs protein. These studies are critical to the development of RVFV attenuated vaccines that have been created so far, as well as the research on novel targets for effective viral inhibitors.

According to the effects of exosomes, it is verified that antiviral autophagy can be induced by IFN signals in RVFV infection. ERK1/2 Akt/mTOR signaling pathway might participate in the antiviral immunity since they have been in anti-tumor immunity (81). Meanwhile, IFN-β also activates caspase-dependent apoptosis, and autophagy could in turn decrease apoptosis to promote cell growth (81). Although IFN-induced innate immunity is TLR-independent, there still exists a Toll receptor-autophagy axis in RVFV infection with Toll-7 and Toll-like receptor adaptor MyD88 (82). Since NSs presents potent suppression of IFN-β transcription and induces the p53 signaling pathway to increase cell apoptosis (83), it is of great significance to discover other independent autophagy-activated systems to restrict viral replication in time. Further research on the precise mechanism in which autophagy is initiated by anti-RVFV innate immune responses and on the intrinsic correlation between IFN-induced autophagy and IFN-related apoptosis during RVFV infection is still needed.

TCF/β-catenin complexes can upregulate the level of IFN-β expression in response to RVFV infection, which is antagonized by the virulence factor NSs. In addition, Wnt/β-catenin signals are shown to regulate the polyamine metabolic pathway in aggressive prostate cancer, reducing the concentrations of citrate and spermine (84). β-catenin signals also promote fatty acid β-oxidation as energy resources for osteoblast metabolism (85). Given that polyamine depletion and fatty acid synthesis inhibition could shut off viral replication and stimulate IFN-induced innate immune responses, it is a promising strategy to target the Wnt/β-catenin pathway and produce a combined action to limit the progress of RVFV infection.

NSs is a key virulence factor of phenuiviruses as an antiviral immune antagonist, but NSs in these viruses have slight differences in anti-immune mechanisms and corresponding cellular effects. Unlike RVFV filamentous NSs in the nucleus, SFTSV, Toscana virus (TOSV) and Uukuniemi virus (UUKV) NSs proteins localize only in the cytoplasm, so they could not directly inhibit host transcription. Besides the unique cytoplasmic granules generated by SFTSV NSs to sequester numerous host factors, TOSV NSs degrades PKR to facilitate viral translation similarly with RVFV, but TOSV NSs could also degrade RIG-I to suppress IFN-β signal activation with its E3 ubiquitin ligase activity (86, 87). NSs of the Punta Toro virus (PTV) can inhibit host transcription, but the nuclear NSs does not form a filamentous structure (88). Sandfly fever virus (SFV) NSs blocks downstream IFN-I signals by inhibiting Jak1 phosphorylation (89). In general, despite the genetic diversity of NSs among different phenuiviruses, it is of great significance to find out their highly conserved IFN-inhibitory activity in immune evasion to drive the development of broad-spectrum drugs and effective vaccines.

From what has been discussed above, most studies have concentrated on how the virus works to counteract the innate immune response, which is the body’s initial and first line of defense. However, RVFV has also developed additional means of attacking various cellular processes, like the cytopathic effects and pro-apoptosis. These methods contribute to viral pathogenicity and should be further investigated in the future.

## Author contributions

LZ conceived the study. XW wrote the first draft. YY and LZ revised the manuscript. XW and YL generated the Figures. All authors read and approved the final manuscript.
